# Sarcomatoid Urothelial Carcinoma With Myxoid Stroma: A Case Report and Diagnostic Approach

**DOI:** 10.7759/cureus.14007

**Published:** 2021-03-20

**Authors:** Rabia Taseer, Tabeer T Ahmed

**Affiliations:** 1 Histopathology, Shiekh Zaid Hospital, Lahore, PAK; 2 Histopathology, Obeid Specialized Hospital, Riyadh, SAU; 3 Pathology, Obeid Specialized Hospital, Riyadh, SAU; 4 Internal Medicine, Combined Military Hospital Lahore Medical College and Institute of Dentistry, Lahore, PAK

**Keywords:** urinary bladder, urothelial carcinoma, sarcomatoid variant, myxoid morphology

## Abstract

Bladder cancer is the seventh most common cancer in the world. Urothelial carcinoma is the most common type of bladder cancer. Other subtypes like squamous cell carcinoma and adenocarcinoma are less common. Urothelial carcinoma has a propensity for divergent differentiation. Sarcomatoid carcinoma is one of the variants of urothelial carcinoma. It is an aggressive tumor that presents at an advanced stage and has a poorer prognosis than conventional urothelial carcinoma. Therefore, identifying this variant histology is important clinically. The sarcomatoid component in sarcomatoid carcinoma can be spindle cell (not otherwise specified), myxoid, pseudoangiosarcomatous, and undifferentiated sarcoma like. Myxoid stroma in sarcomatoid urothelial carcinoma has been described but reported very rarely. We present a case of sarcomatoid urothelial carcinoma with myxoid stroma along with a review of the diagnostic approach to myxoid spindle cell lesions of the urinary bladder.

## Introduction

Bladder cancer is the seventh most common cancer in the world [1]. It is three to four times more common in men than in women and the median age of a diagnosis is 65-70 years [2]. The most common type of bladder cancer is urothelial carcinoma. Other subtypes like squamous cell carcinoma and adenocarcinoma are less common [3]. Urothelial carcinoma has a propensity for divergent differentiation. Squamous, glandular, micropapillary, nested, plasmacytoid, lymphoepithelioma-like, and sarcomatoid are prominent examples [4]. Sarcomatoid carcinoma was initially described as carcinosarcoma by Dent [5]. The recent World Health Organization classification endorses the term sarcomatoid carcinoma. It accounts for 0.6% of all bladder tumors [6]. It is an aggressive tumor that presents at an advanced stage and confers a much poorer prognosis than conventional urothelial carcinoma. Therefore, identifying this variant histology is important clinically [7,8]. The sarcomatoid component in sarcomatoid carcinoma can present with variable morphology. They can be spindle cells (not otherwise specified), myxoid, pseudoangiosarcomatous, and malignant fibrous histiocytoma-like undifferentiated features. A myxoid subtype of sarcomatoid carcinoma is associated with poor prognosis [9]. It has been reported very rarely. We present a case of sarcomatoid urothelial carcinoma with myxoid stroma, in a 56-year-old male patient. In addition, we include a discussion of the diagnostic approach to myxoid spindle cell lesions of the urinary bladder.

## Case presentation

A 56-year-old male patient presented to the urology department with severe hematuria. He had severe anemia with a hemoglobin of 5.6 g/dl. He was given three units of blood to improve hemoglobin. On ultrasonography, there was an ill-defined hypoechoic lesion in the right wall of the bladder measuring 25 mm in maximum dimension. Computed tomogram showed a mural soft tissue heterogeneously enhanced mass measuring 44 mm.

**Figure 1:**
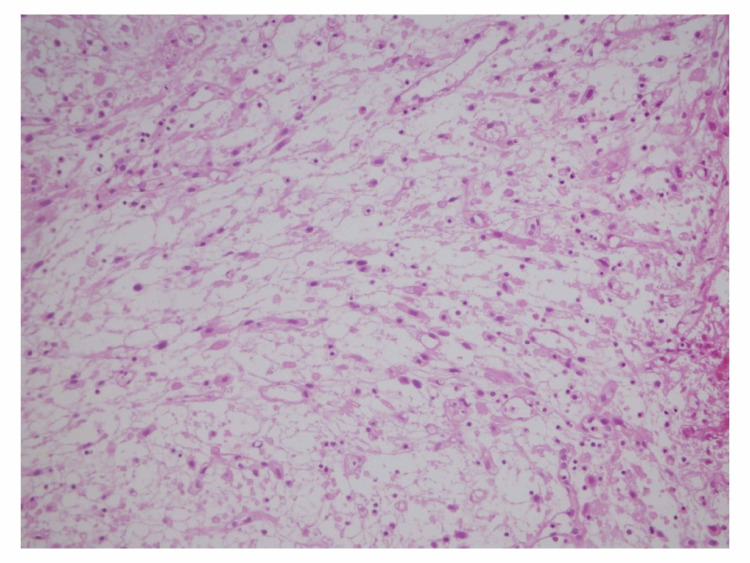
Loosely arranged spindle cells with myxoid stroma and thin capillaries. Medium power (20×).

**Figure 2:**
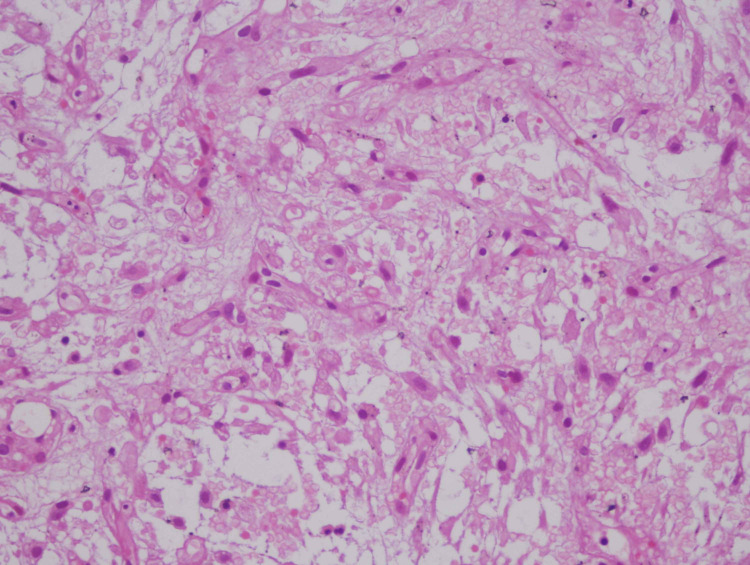
Loosely arranged spindle cells on a myxoid background. High power (40×).

**Figure 3:**
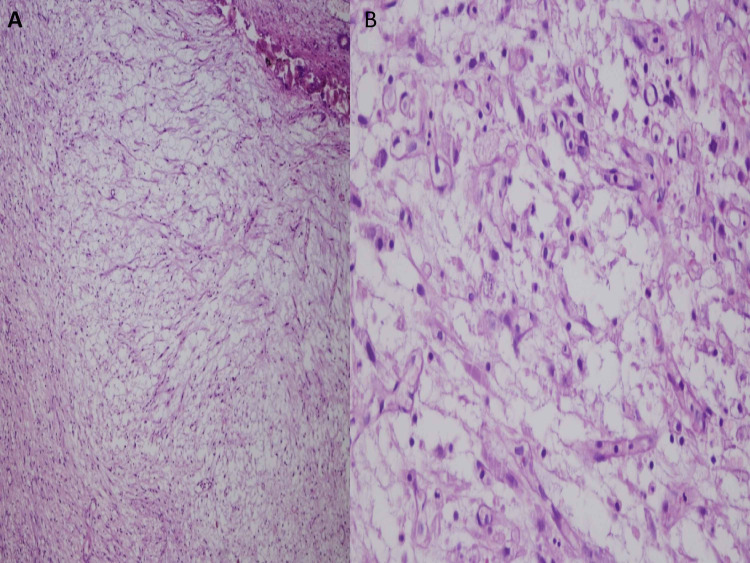
Hematoxylin and eosin stain - section of bladder mass showing spindle to polygonal cells arranged on a loose myxoid stroma. (A) Medium power (20×) and (B) high power (40×).

**Figure 4:**
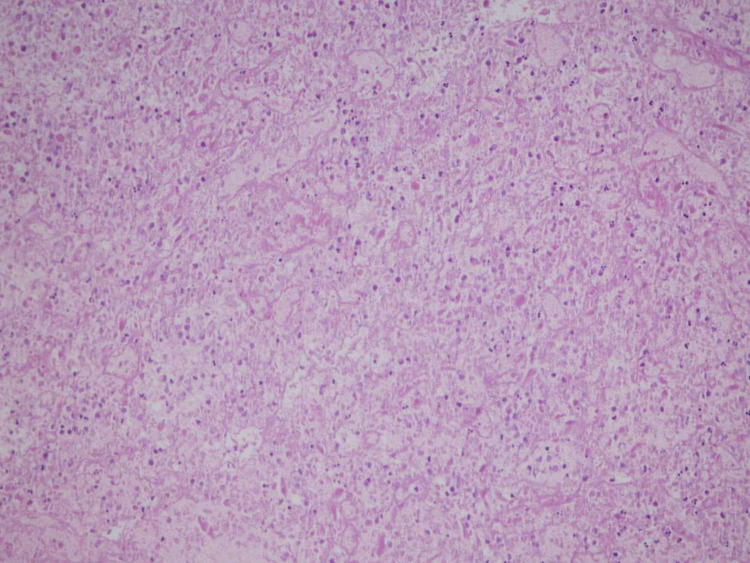
Hematoxylin and eosin stain. Bladder mass biopsy - a large area of necrosis. Medium power (20×).

Once hemoglobin levels improved, a cystoscopy was performed. There was a polypoid bladder mass in the right wall. Multiple biopsy fragments were taken. The histopathology department received tissue fragments weighing around 30 g. The entire specimen was submitted for evaluation. Hematoxylin and eosin-stained sections revealed a spindle cell lesion/neoplasm, showing marked myxoid stromal changes. There was significant nuclear pleomorphism, but only very occasional mitoses were seen (Figures 1 and 2). At places, the cells had epithelioid morphology. There were no definite sheets or cohesive clusters (Figure 3). There was a mixed inflammatory infiltrate and large areas of necrosis were seen (Figure 4). The differential diagnoses included sarcomatoid carcinoma, myxoid leiomyosarcoma, and inflammatory myofibroblastic tumor.

**Figure 5:**
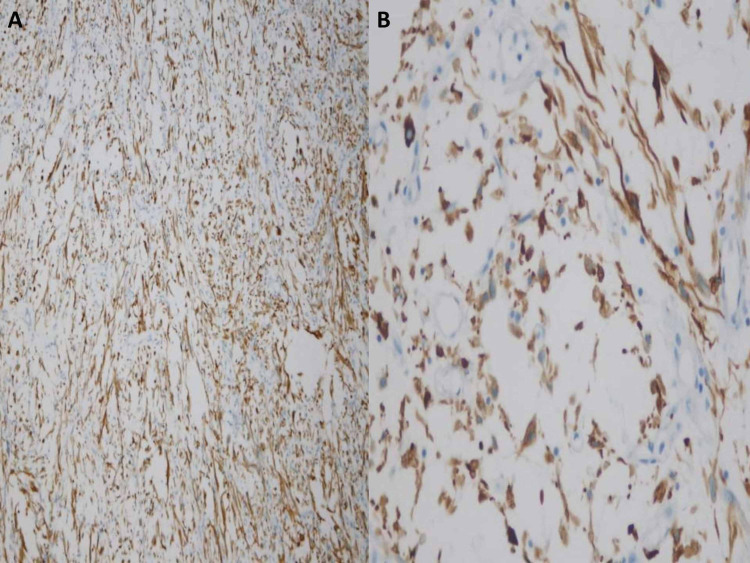
Pan cytokeratin immunostaining; the spindle cells are stained diffusely: (A) 20× and (B) 40×.

**Figure 6:**
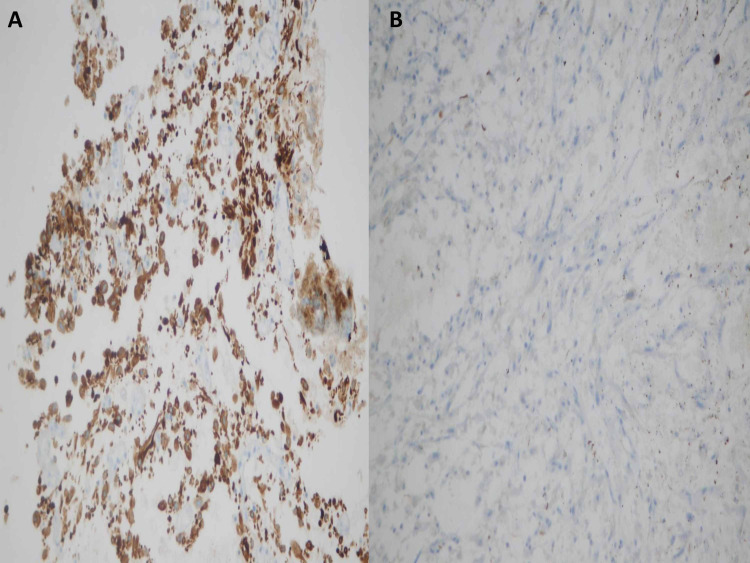
Immunostains, bladder mass biopsy. (A) Spindle cells are positive for CAM 5.2 and (B) negative for desmin.

**Figure 7:**
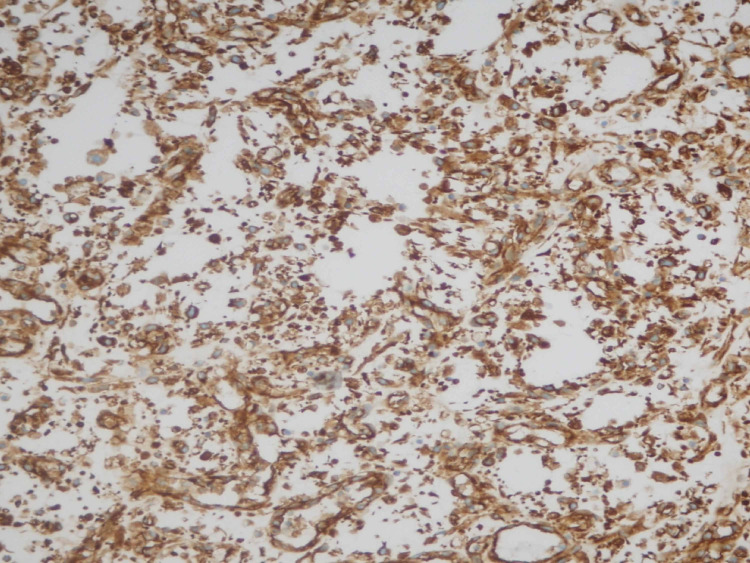
Immunostain vimentin. Diffusely positive in malignant cells. Vimentin is considered a marker of epithelial-mesenchymal transition.

**Figure 8:**
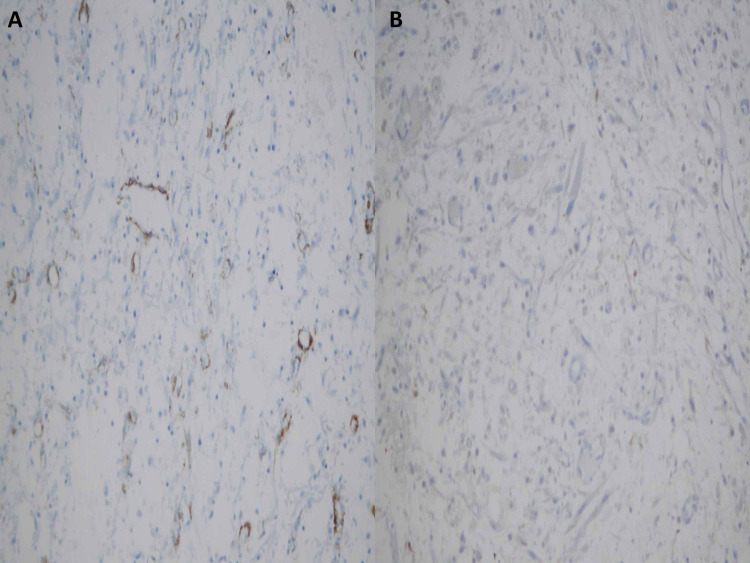
Negative immunostains. (A) Smooth muscle actin and (B) anaplastic lymphoma kinase 1 protein.

On immunohistochemistry, the neoplastic spindle cells were strongly positive for Pan-Cytokeratin, CAM5.2 (Figures 5 and 6) CK 7, CK20, and vimentin (Figure 7). Ki67 index was high in hot spots. The tumor cells were negative for smooth muscle actin (SMA), desmin, calponin, caldesmon, anaplastic lymphoma kinase (ALK1) protein, and CD34 ruling out the differential diagnosis of leiomyosarcoma with myxoid stroma and Inflammatory myofibroblastic tumor (Figure 8). The tumor cells failed to express GATA3. CDX2 was negative ruling out the possibility of involvement of bladder wall by mucinous carcinoma of the colon. Sarcomatoid prostatic carcinoma can also extend to involve the bladder wall. Positive CK7 and CK20 with negative prostate-specific antigen (PSA) ruled out this possibility. A final diagnosis of a high-grade urothelial carcinoma (grade 3) showing a spindle cell morphology (sarcomatoid carcinoma) was made. The tumor showed focal muscle invasion stage pT2a (at least). No lymphovascular or perineural invasion was seen. The patient was referred to a specialized oncology center for further management.

## Discussion

Bladder cancer is the seventh most common cancer in the world [1]. It is three to four times more common in men than in women and the median age at diagnosis is 65-70 years [2]. The most common type of bladder cancer is urothelial carcinoma. Other subtypes like squamous cell carcinoma and adenocarcinoma are less common [3]. Urothelial carcinoma can show divergent differentiation. Squamous, glandular, micropapillary, nested, plasmacytoid, lymphoepithelioma-like, and sarcomatoid carcinoma are prominent examples [4]. Sarcomatoid carcinoma was initially described as carcinosarcoma by Dent [5]. The recent World Health Organization classification endorses the term sarcomatoid carcinoma. It accounts for 0.6% of all bladder tumors [6].

Morphologically sarcomatoid carcinoma shows a mixture of conventional urothelial carcinoma or glandular or any epithelial component with variable degrees of differentiation mixed with the sarcomatous component. The epithelial component may be scarce or even not identified in some cases, so the diagnosis of primary sarcoma in the urinary bladder should be made with caution in transurethral resection specimens [7,8]. The sarcomatoid component can constitute 20% to 100% of the tumor volume. On average it constitutes 65% of total tumor volume. It can present with variable morphology including spindle cell (not otherwise specified), myxoid, pseudoangiosarcomatous, and malignant fibrous histiocytoma-like undifferentiated features [9]. Myxoid morphology has been described but has rarely been reported. Our case comprised mainly of sarcomatous components; there was no epithelial element. In addition, the spindle cells were embedded in an abundant myxoid stroma.

Sarcomatoid urothelial carcinoma can show heterologous elements like osteosarcoma, rhabdomyosarcoma, and chondrosarcoma. The presence of heterologous elements can be associated with a worse prognosis [6]. The prominent morphological feature in our case was spindle to epithelioid cells arranged in a loose myxoid stroma. No heterologous element was seen in this specimen.

According to Manini and López, it is important to know different unusual morphological features of urothelial carcinoma. An immunohistochemical study is a major tool in establishing a diagnosis. Molecular analysis can be useful where applicable [10]. In spindle cell lesions of the bladder, especially those with myxoid stromal features, sarcomatoid urothelial carcinoma, and leiomyosarcoma top the list of differential diagnoses followed by the inflammatory myofibroblastic tumor. Leiomyosarcoma is the commonest sarcoma in the urinary bladder accounting for 1% of total bladder malignancies. It can show myxoid stromal changes [11]. Leiomyosarcoma commonly presents as a high-grade lesion in an advanced stage. The prognosis is generally poor [12]. On the other hand, an inflammatory myofibroblastic tumor of the genitourinary tract is a neoplasm of uncertain malignant potential [13]. Morphologically, it shows loose stellate cells with a myxoid background containing scattered inflammatory cells. Detection of ALK 1 protein and ALK gene rearrangements are useful in distinguishing inflammatory myofibroblastic tumors from spindle cell malignancies in the urinary bladder. Inflammatory myofibroblastic tumors are positive for SMA and desmin. It can also show aberrant expression of cytokeratins [14]. Therefore, it is very important, that a wide immunopanel is used in case of spindle cell lesions of the urinary bladder.

In a detailed paper, Westfall et al. have illustrated that within the context of morphology, an immunohistochemical panel composed of broad-spectrum antibodies to cytokeratin as well as antibodies to SMA, ALK, p63, and CK 5/6 should be used. According to them, a combination of Pankeratin, SMA, and ALK positivity favors inflammatory myofibroblastic tumor; expression of several cytokeratins, especially CK 34betaE12 and CK 5/6 with p63 favors sarcomatoid carcinoma. SMA positivity with the overall absence of other markers favors leiomyosarcoma [15]. The present case showed positivity for Pancytokeratin, CK 7, CK20, and CAM 5.2. Immunohistochemistry for ALK1 protein, CD34, SMA, calponin, and desmin was negative ruling out the differential diagnosis of inflammatory myofibroblastic tumor and leiomyosarcoma.

Vimentin is considered a marker of epithelial-mesenchymal transition. It is expressed in 100% of cases of sarcomatoid urothelial carcinoma [9]. GATA-3 expression is present in 70% of conventional bladder urothelial carcinoma, but its expression differs among variants of urothelial carcinoma. Sarcomatoid variant shows GATA3 positivity in only 16% of cases [16]. Our case showed a strong diffuse expression of vimentin but there was no GATA3 expression.

Lastly, colonic adenocarcinoma with mucinous stroma can also invade the bladder wall and present as a poorly differentiated malignant lesion. Immunohistochemistry can again be helpful in these cases. Co-expression of CK7 and CK20 is seen in bladder carcinoma and it is negative for CDX2. On the other hand, colon carcinoma is negative for CK7 and positive for CK20 and CDX2 [17]. Malignant cells were positive for CK7, CK2O and negative for CDX2 in our case. Sarcomatoid prostatic carcinoma was also considered in the differential. CK20 and CK7 expressions ruled out this possibility. PSA was also negative. The final diagnosis of sarcomatoid urothelial carcinoma was made.

In a comprehensive study by Malla et al., it was concluded that patients with sarcomatoid urothelial carcinoma present with a high histologic grade, advanced-stage disease, and a poor prognosis. They further reiterated that sarcomatoid urothelial carcinoma is not as rare as previously thought and the patients should be referred to a center with experience of this disease [18]. There is no standardized clinical management for sarcomatoid carcinoma, and adjuvant therapy can vary in different institutions [7].

## Conclusions

Sarcomatoid carcinoma with myxoid stroma is a recognized but rare example of divergent differentiation in urothelial carcinoma. It is important to recognize this pattern as clinically sarcomatoid carcinoma has been associated with aggressive behavior and poor prognosis. Immunohistochemistry is the most important tool for a definite diagnosis of this entity. Owing to the difference in prognosis and outcomes in case of different entities showing spindle cell morphology with myxoid stroma, it is important that the transurethral resection specimens are evaluated carefully with thorough sampling and use of immunohistochemistry.
